# The impact of drug eluting stents on the co-release of interleukin-6 in patients with stable angina

**DOI:** 10.21542/gcsp.2024.53

**Published:** 2024-12-31

**Authors:** Zeyad Albadri, Reem Almousali, Souhila Abdulla

**Affiliations:** 1 Department of Clinical and Experimental Medicine, Faculty of Health Sciences, Linköping University, Linköping, Sweden; 2 Medical Department, Bra Liv Medical Centres, Huskvarna, Sweden; 3 Department of Statistics, Cihan University, Erbil, Iraq

## Abstract

**Background**: Drug-eluting stents represent an important revolution in the treatment of coronary artery disease. However, the mechanical trauma of stent deployment can trigger vascular injury and inflammatory cytokine cascades, potentially precipitating in-stent restenosis. The release of cytokines is not firmly established in patients with stable angina.

**Aims**: To evaluate the acute effect of drug-eluting stents on the acute inflammatory response in patients with stable angina by quantifying interleukin-6 levels.

**Methods and materials**: A single-centre exploratory study was conducted on 13 patients with stable angina undergoing elective angioplasty. Arterial blood samples were collected before angioplasty and directly after angioplasty. Additionally, venous blood samples were collected 24 h post-angioplasty. Concentrations of interleukin-6 were analysed to assess changes in the acute inflammatory response.

**Results**: IL-6 concentration significantly increased immediately after stent placement (5.95 ± 0.49 pg/ml, *p*  =   < 0.05) and 24 h post-stent placement (8.96 ± 1.16 pg/ml, *p*  =   < 0.01). Gender-based analysis indicated significant increases in IL-6 levels in males post-stent placement (5.94 ± 0.08 pg/ml, *p*  =   < 0.05) and 24 h post-stent placement (7.27 ± 0.56 pg/ml, *p*  =   < 0.01). Statin medication significantly reduced IL-6 expression in patients at baseline (3.27 ± 0.71 pg/ml versus 5.44 ± 0.18 pg/ml, *p*  =   < 0.05), but this distinction diminished rapidly after stent insertion, resulting in comparable IL-6 levels at 24 h post-stent insertion in both groups.

**Conclusion**: Interleukin-6 levels markedly increased immediately after coronary stenting, suggesting its role as an early initiator of the inflammatory response to coronary stenting. While limited by sample size, it lays the groundwork for larger, more comprehensive research to optimize drug-eluting stents outcomes and inflammatory management.

## Background

The evolution of interventional cardiology has been marked by continuous advancements in techniques and technologies aimed at improving the outcomes of angioplasty^1^. However, long-term complications such as restenosis remain an important limitation to percutaneous coronary intervention (PCI)^2^. Drug-eluting stents (DES) are coated with potent antimitotic agents that inhibit smooth muscle proliferation and matrix growth, thus preventing neointimal proliferation and restenosis^3^. In addition to inhibiting smooth muscle cell proliferation, DES inhibit the local inflammatory response in the acute phase after implantation, which also contributes to the prevention of restenosis^3–5^. Furthermore, stent implantation elicits the release of various cytokines leading to systemic inflammatory responses whose intensity and magnitude are associated with a worse clinical outcome and increased risk of stent restenosis^6–8^. However, the predictive value of baseline inflammatory biomarkers after DES placement is controversial.

The aim of this study is to investigate the acute inflammatory response in patients with stable angina by measuring the plasma levels of interleukin-6 (IL-6), a pro-inflammatory cytokine before and after DES implantation. Additionally, we assessed the effect of statin treatment on the expression of IL-6 levels and if there was a possible gender difference in the IL-6 levels release before and after DES implantation.

## Material and Methods

### Study population

Thirteen patients with a history of stable angina were included in this study. All patients underwent elective PCI with stent implantation with at least one DES for previously untreated lesions in a single coronary vessel. Patients were excluded if they had myocardial infarction within the last month, by-pass graft, unstable angina, received radiotherapy or chemotherapy recently, or in stent restenosis or total coronary occlusion or intolerance to aspirin, heparin or clopidogrel. The local institutional review board approved the study protocol, and written informed consent was obtained from each patient.

### Interventional procedure

All patients received oral aspirin, clopidogrel and intravenous heparin boluses were administered before and after PCI to increase the activated partial thromboplastin time by 1.5 to 2 times the normal control values. Selective PCI was performed and the most accurate evaluation of the stenotic lesion made according to the standard intervention protocol. The choice of specific DES was left to the operator’s discretion.

### Blood sample collection

Blood samples were collected from the arterial access sheath at the time of the PCI and immediately after placement of the coronary stent. Subsequently, blood samples were obtained by peripheral venepuncture from the Brachial vein at 24 h post PCI. Venous samples were collected in heparin-coated tubes. Serum was prepared by centrifugation to isolate the plasma needed for assay within 1 hr of sample collection and samples were stored at −80 °C in 0.5 ml aliquots until assay was performed.

### Measurement of cytokine

Serum IL-6 concentrations were measured using enzyme-linked immunosorbent assay (ELISA) using 96-well specific antibodies plate (Nunc Immuno, Denmark). They were incubated overnight at room temperature with capture antibody (R & D Systems Minneapolis, USA). The assay was conducted according to the ELISA kit manufacturer’s instructions. Each determination was performed in triplicate. A standard curve was performed by plotting the standards and IL-6 concentrations were expressed as pictograms per millilitre (pg/ml)^9^.

### Statistical analysis

The primary aim was to investigate IL- 6 concentrations of patients with stable angina before and after DES implantation. Values were expressed as mean ± standard deviation. Statistical analysis was performed using the nonparametric Kruskal-Wallis test, the Mann–Whitney U test, or the Wilcoxon signed rank test. Determinations of the distributions of IL-6 values were assessed at each time point; repeated analysis of variance was used to determine if there was significant increase in these markers immediately and 24 h post-angioplasty. Gender and statin treatment comparisons were analysed using unpaired Student’s t tests for continuous variables and Chi-square tests for categorical variables. A *p* value below 0.05 was considered statistically significant.

## Results

### Patient characteristics

Thirteen patients (eight males and five females) all diagnosed with stable angina and implanted with a DES were recruited for this study. The average age of the subjects was 62 ± 4.1 years. Detailed baseline characteristics of the patients are presented in Table 1.

**Table 1:** Baseline characteristics of the patients.

Patients Characteristics	
No. of patients	13
Age, years	62 ± 4.1
Male gender, n (%)	8 (61%)
Diabetes, n (%)	4 (31% )
Hypertension, n (%)	5 (38%)
Dyslipidaemia, n (%)	6 (46%)
Instable Angina, n (%)	13 (100%)
**Medications**	
Statins, n (%)	6 (46%)
Beta-blockers, n (%)	9 (69%)
ACEIs/ARB, n (%)	10 (77%)
Nitrates, n (%)	12 (92%)
Smoking, n (%)	6 (46%)

**Notes.**

ACEI angiotensin-converting enzyme inhibitors ARB angiotensin receptor blockers

### IL-6 expression

**Figure 1.**
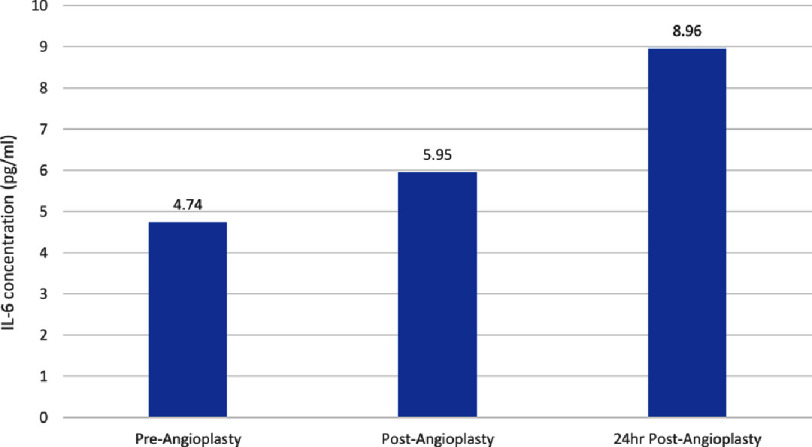
Plasma levels of interleukin-6 at baseline, immediately after stenting, and 24 h after drug eluting stent implantation. IL, interleukin.

Our investigation revealed a significant increase in IL-6 concentration immediately after stent placement (5.95 ± 0.49 pg/ml, *p*  =   < 0.05) and 24 h post-stent placement (8.96 ± 1.16 pg/ml, *p*  =   < 0.01) from baseline levels (4.74 ± 0.52 pg/ml). Figure 1 summarizes these results before and after stent implantation.

### Gender impact on IL-6 expression

**Figure 2.**
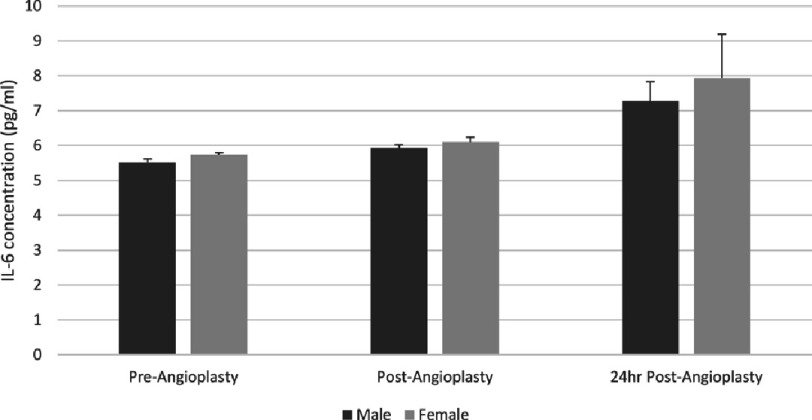
Gender impact on serum levels of interleukin-6 at baseline, immediately after stenting, and 24 h after stenting. IL, interleukin.

There were no significant differences in IL-6 between the genders. However, male patients showed significantly increased IL-6 levels post-stent placement (5.94 ± 0.08 pg/ml, *p*  =   < 0.05) and 24 h post-stent placement (7.27 ± 0.56 pg/ml, *p*  =   < 0.01) from baseline levels (5.52 ± 0.09 pg/ml). While in female patients, IL-6 levels increased but not as significantly post-angioplasty (6.10 ± 0.14 pg/ml, *p* = >0.05) and 24 h post-angioplasty (7.93 ± 1.26 pg/ml, *p* = >0.05) from baseline values (5.73 ± 0.07 pg/ml) as shown in Figure 2.

### Statin’s effect on IL-6 expression

**Figure 3.**
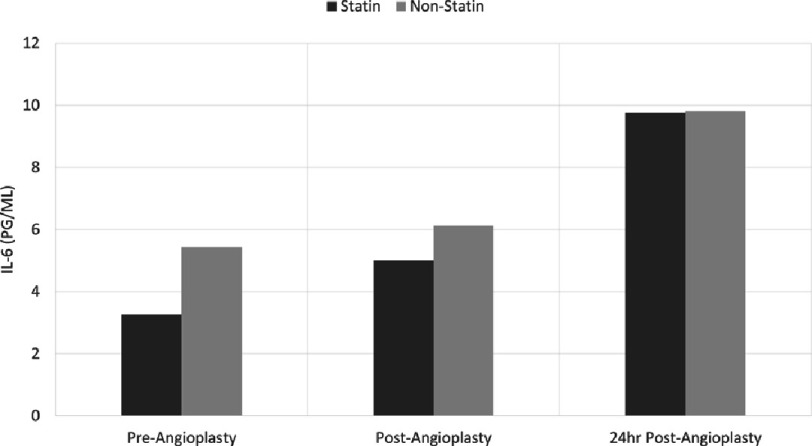
Statin impact on serum levels of interleukin-6 at baseline, immediately after stenting, and 24 h after stenting. IL, interleukin.

The impact of statins on IL-6 expression was significantly reduced in patients receiving statin medication compared to those patients than in patients who were not treated with statins at baseline (3.27 ± 0.71 pg/ml *versus* 5.44 ± 0.18 pg/ml, *p*  =   < 0.05 respectively). However, this distinction diminishes rapidly after stent insertion, resulting in comparable levels of IL-6 at 24 h post- stent insertion in both groups (Figure 3).

## Discussion

This study investigated changes in IL-6 serum levels before and after coronary stenting in a carefully selected population only including patients with stable angina, demonstrating an increase in circulating IL-6 plasma levels immediately and 24 h after stent implantation. The observed elevation in IL-6 levels suggests its role as an early inflammatory mediator and potential biomarker of the acute inflammatory response following stent deployment.

IL-6 is involved in a variety of physiological functions ultimately leading to macrophage activation, platelet aggregation, and stimulation of matrix degrading enzymes. An increase in circulating concentrations of IL-6 and of its hepatic by-product C reactive protein (CRP) has been reported in unstable angina^10^. The release of high concentrations of IL-6 and CRP after PCI are considered markers of poor prognosis, probably related to the intensity of plaque inflammation^11^.

Our findings align with previous studies, indicating that drug-eluting stents do not reduce the levels of acute phase cytokine markers IL-6 in the immediate post PCI period^12,13^. A study conducted in the United States reported significant increases in IL-6 plasma levels 1 h after PCI in patients with^11^. Furthermore, Aggarwal et al. demonstrated that IL-6 increases after PCI in patient undergoing coronary stenting^14^. Other studies have demonstrated a relationship between the increase of IL-6 levels and restenosis after PCI^15^. Liuzzo and colleagues demonstrated that IL-6 increased 6 h after PCI in patients with unstable angina pectoris^16^. Our findings demonstrate that IL-6 increases even earlier within the first hour after stent implantation which is consistent with an increase in IL-6 in the first hour after injection of endotoxin in healthy volunteers^17^. These results indicate that DES have no immediate effect on reducing the level of IL-6 produced in response to the vascular injury caused by stenting.

Gender-based analysis reveals significant IL-6 increases in male subjects, both post-stent placement and 24 h later, while female subjects show an increase that does not reach statistical significance. The mechanism of this gender-related response is difficult to determine within this study. Multiple covariates such as genetic factors, comorbidities, body mass index, serum creatinine, platelet count and individual differences in drug metabolism may influence the effectiveness of these stents in modulating the inflammatory response^18^. Furthermore, menopausal state and hormonal replacement therapy may have important effects on the inflammatory response^19,20^. This study identifies gender as a potentially important variable in the inflammatory response to coronary stenting however; this needs to be confirmed in a larger study sample.

Hyperlipidaemia significantly increases the risk for cardiovascular diseases. Statins exhibit pleiotropic effects that contribute to the reduction of cardiovascular events. Besides lowering serum cholesterol, they have shown anti-inflammatory properties^21^; their beneficial effects in patients undergoing stent deployment have been documented in many clinical studies^22,23^.

The study demonstrates that patients who received statin treatment before the procedure exhibited lower concentrations of IL-6 compared to those who did not receive statins, with a notable difference between subjects on pre-procedure statins (baseline: 3.27 ± 0.71 pg/ml) and those without statin therapy (baseline: 5.44 ± 0.18 pg/ml). No significant differences were observed post-procedure or after 24 h. These results align with a study by Inoue et al., indicating that statins suppress IL-6 expression in human vascular smooth muscle cells, endothelial cells, and macrophages^24^. Studies have demonstrated that statins suppress both the expression of soluble intercellular adhesion molecule-1 in macrophages and the lipopolysaccharide-induced production of IL-6 and tumor necrosis factor-*α* by monocytes and macrophages^25,26^. Our data suggests that statins might act as cytokine suppressive therapy that could inhibit further cardiovascular damage.

The significant increase of IL-6 levels observed in this study indicates the high sensitivity of this cytokine for detecting early inflammatory response after PCI. Our findings underline the possible advantage of screening for IL-6 plasma levels and the use of selective anti-inflammatory treatment to reduce the risk of restenosis. While this may suggest a limited impact on the acute inflammatory response, it is crucial to note that these results do not necessarily contradict potential long-term benefits. The lack of an immediate reduction in acute inflammatory markers suggests that the anti-inflammatory properties of DES may not manifest in the early stages following PCI. This raises questions about the timeline and mechanisms through which these stents exert their therapeutic effects.

As we reflect on our findings, it is essential to highlight the limitations of our study. The most important limitation of our study is the small sample size performed in a single-center that focuses on the immediate post procedure time points may not capture the full spectrum of the long-term inflammatory response. Although we considered the total length or volume of stents by the addition of several small stents, a longer single stent could affect cytokine secretion differently which has not been addressed. Stent length/volume may be extensively assessed in future studies with more subtle criteria to evaluate the extent of cytokine secretion. Future studies with larger cohorts and extended follow-up periods should aim to address these limitations and provide a more comprehensive understanding of the temporal dynamics of inflammation in response to DES.

## Conclusions

Our findings corroborate existing literature on the acute inflammatory response to DES implantation. The significant IL-6 rise highlights a crucial inflammatory phase immediately after stenting, unaffected by statins in the acute phase. While our small sample limits generalizability, the results are consistent with larger studies, suggesting reproducibility. The absence of a control group and the single-center design are acknowledged limitations. Future studies should include multi-center data, extended follow-up, advanced models accounting for repeated measures and comparative cohorts to contextualize IL-6’s role in long-term outcomes.

## Conflicts of Interest

The authors have declared they have sno conflict of interest.
